# Choroidal granuloma caused by *Mycobacterium Fortuitum*

**DOI:** 10.1186/s40942-019-0185-8

**Published:** 2019-10-14

**Authors:** João Pinto da Silva Neto, Kyra Nhayanna Coutinho Machado, Luiz Roisman

**Affiliations:** 1 Hospital Federal da Lagoa, Rio de Janeiro, Brazil; 2 Federal University of São Paulo, São Paulo, Brazil

**Keywords:** *Mycobacteria Fortuitum*, Choroidal granuloma, Mycobacteria

## Abstract

**Background:**

To report a case of a chronic steroid user male patient who developed local abscesses caused by *M. fortuitum* and concomitant asymptomatic choroidal granuloma.

**Case presentation:**

A 37-year-old african-american male with history of use of anabolic drugs and intramuscular mineral oil injections in the upper and lower limbs for 15 years for muscular hypertrophy. He developed intramuscular abscesses with systemic infection, sub-retinal lesions in both eyes and alterations in cerebrospinal fluid suggestive of mycobacteria. Considering these findings, empirical treatment for tuberculosis was started, without success. After several negative cultures of the material drained from the abscesses, finally one of the cultures isolated the agent *Mycobacterium Fortuitum.* Proper treatment for atypical mycobacteria was initiated with clinical and laboratory improvement. After 6 months the sub-retinal lesions regressed.

**Conclusions:**

A typical choroidal granuloma caused by *M. Fortuitum* is a rare presentation of the infection and our report showed a good outcome with proper treatment.

## Background

The genus *Mycobacterium* includes more than 50 species along with typical (tubercular) and atypical mycobacteria (non-tubercular) [1]. There are four groups of nontuberculous mycobacteria (NTM). The most common species causing ocular NTM infections are *M. chelonae*, *M. abscessus*, and *M. fortuitum* [2]. Rapidly growing mycobacteria are associated with ocular infections including canaliculitis, dacryocystitis, scleritis, keratitis, uveitis, orbital cellulitis, endophthalmitis and panophthalmitis [2–4].

Immunocompromised states (e.g. use of steroids) are risk factors for development of opportunistic infections, including NTM infection [1–5].

We report a case of a male patient whose chronic steroid use and mineral oil injections for the purpose of muscle hypertrophy led to the development of local abscess by *M. fortuitum* and concomitant asymptomatic choroidal granuloma.

## Case presentation

A 37-year-old african-american male was referred to the endocrinology department of the Federal Hospital of Lagoa for evaluation of adrenal insufficiency due to chronic and irregular use of steroids (Prednisone 20 to 40 mg/day) for 10 years. He had history of use of anabolic drugs and intramuscular mineral oil (hydrogel) injections in the upper and lower limbs for 15 years for muscle hypertrophy. Three months prior to the referral he developed intense headache, fever, night sweats, weight loss and limb pain. No other systemic comorbidities.

During hospitalization, intramuscular purulent collections were diagnosed by imaging tests and he presented daily fever (maximum of 38 °C or 100 °F). Treatment with piperacillin*/*tazobactam 2.25 g IV q6hr and vancomycin 500 mg IV q6hr was initiated and laboratory tests collected. The blood tests showed 11,900 leukocytes (90% neutrophils), high level of C reactive protein, negative blood culture, culture of the intramuscular purulent material negative, negative serology for HIV and syphilis. Chest and cranial computed tomography scans were normal. The cerebrospinal fluid showed the presence of 330 cells (60% mononuclear), 239 mg/dL proteins, 49 mg/dL glucose, negative Nanquin test, negative latex fixation test, negative bacterioscopy, negative cytomegalovirus serology. The polymerase chain reaction (PCR) result for Koch’s bacillus was in process at this time. Drainage of right thigh abscess was performed and material sent to culture.

**Fig. 1:**
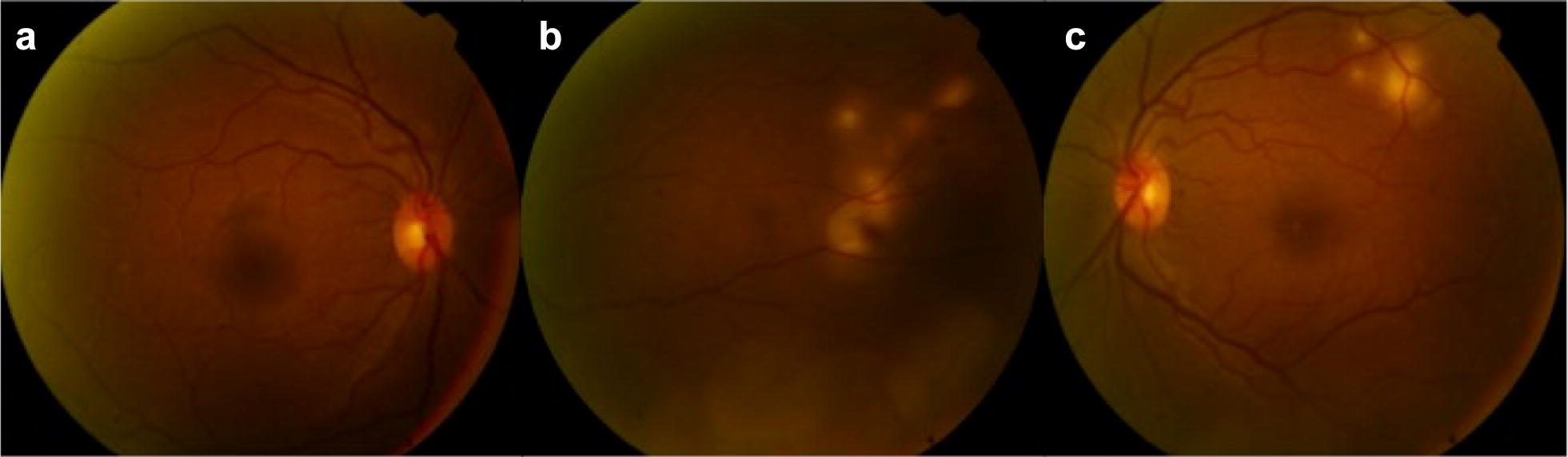
Fundus color picture showing (**a**) an unremarkable posterior pole and (**b**) subretinal, elevated, rounded, yellowish lesion in the nasal region in the right eye. **c** Similar lesion superior to the macula were found in the left eye

Ophthalmology Service was then requested for evaluation. Visual acuity was 20/20 in both eyes, anterior biomicroscopy and intra-ocular pressure were normal. Fundus examination revealed subretinal, elevated, rounded, yellowish lesions in the nasal region in the right eye and superior to the macula in the left eye (Fig. 1).

**Fig. 2:**
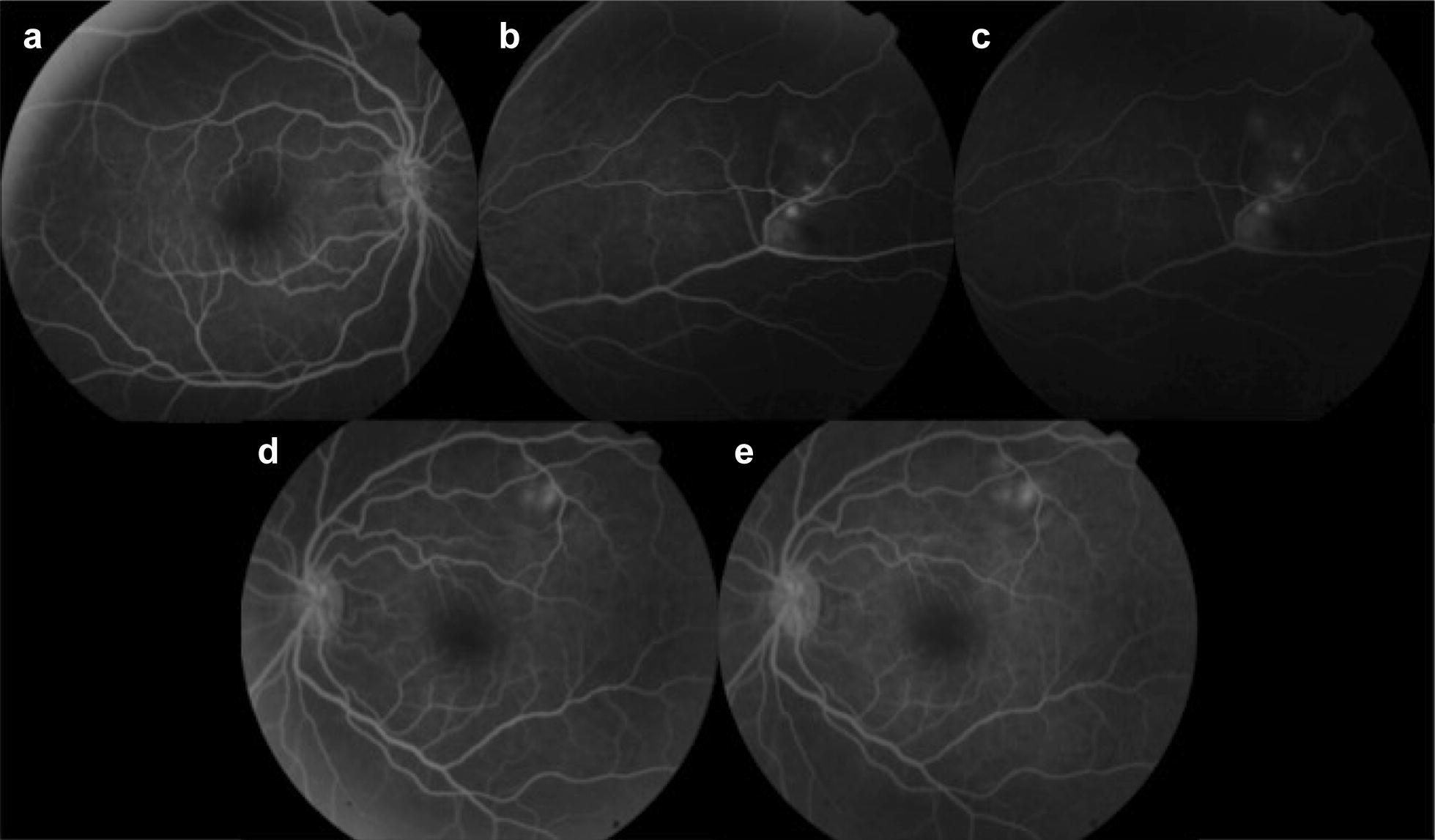
Fluorescein angiography of the right (**a**–**c**) and left eye (**d**, **e**). The subretinal lesions revealed punctate leakage in both eyes

**Fig. 3:**
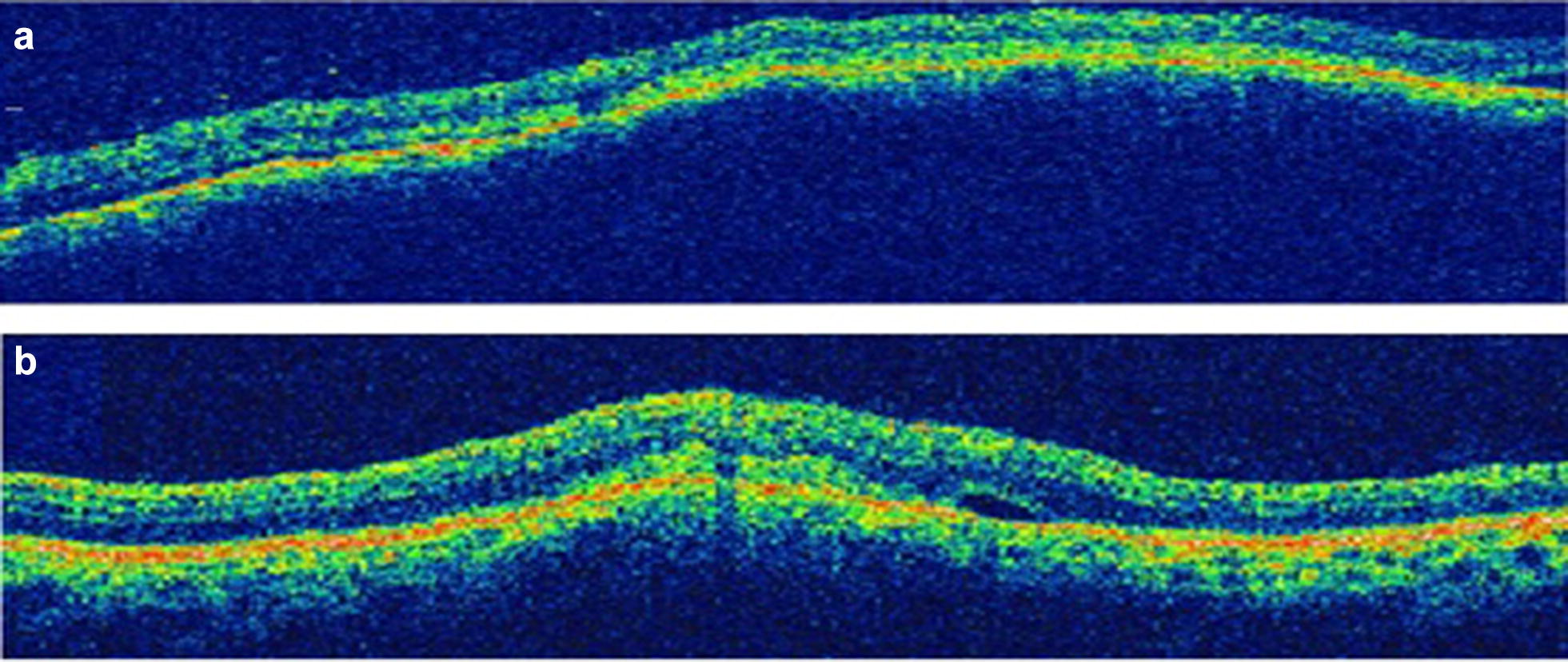
Optical coherence tomography of the lesions of the right (**a**) and left (**b**) eye showing choroidal lesions causing elevation of the retinal pigment epithelium and subretinal fluid

On fluorescein angiography (FA), these lesions showed punctate leakage (Fig. 2). The optical coherence tomography (OCT) revealed choroidal lesions causing elevation of the retinal pigment epithelium (RPE) and subretinal fluid (Fig. 3).

Considering systemic symptoms, suggestive laboratory and retinal lesions, the hypothesis of systemic tuberculosis with choroidal granuloma was assumed. After 14 days of treatment with piperacillin/tazobactam and vancomycin without clinical or laboratory response, the therapy was switched to rifampicin, isoniazid, pyrazinamide and ethambutol. At this point, several cultures of the material drained from the abscess were negative.

After 4 weeks of treatment for tuberculosis, the patient maintained the symptoms and no improvement of laboratory tests. The possibility of fast growing atypical mycobacteria was considered and the empirical treatment for mycobacteria with amikacin and clarithromycin was added to previous tuberculosis treatment.

Posteriorly to the change on the treatment, the patient presented clinical improvement and leukocytes progressively reduced from 16,500 to 7800 after 2 days. Then, one of the cultures collected from the left thigh confirmed the growth of non-tuberculous mycobacteria and the agent *Mycobacterium Fortuitum* was isolated. It was susceptible to the following antibiotics: amikacin, ciprofloxacin, doxycycline and moxifloxacin. At this time, the antibiotic therapy was replaced by doxycycline 200 mg/day and ciprofloxacin 1 g/day, oral administration.

**Fig. 4:**
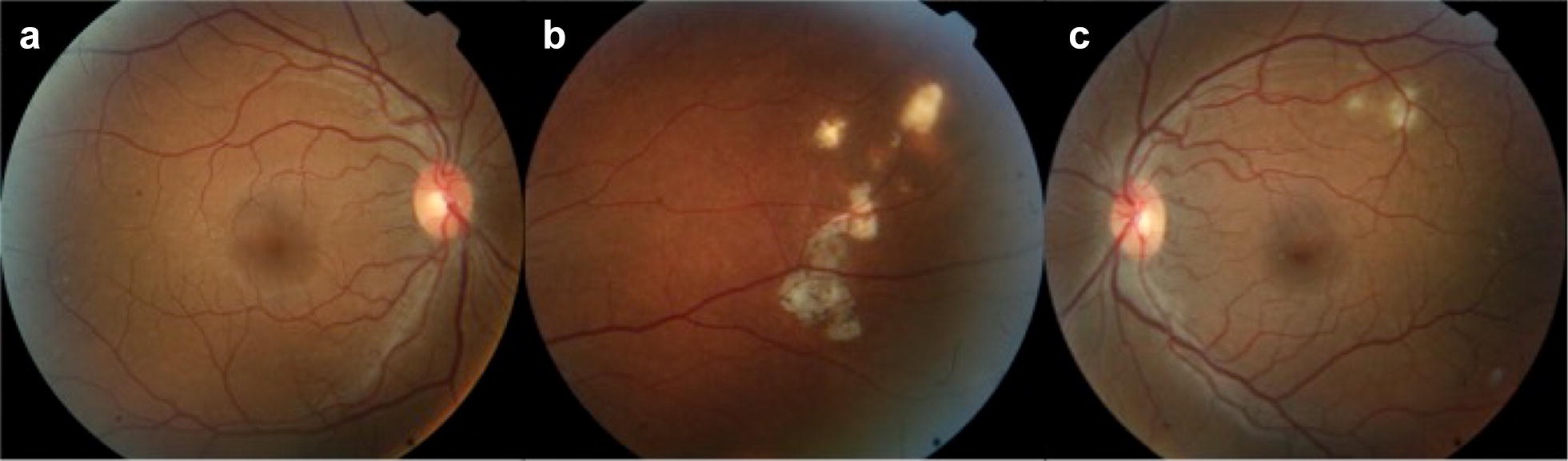
Fundus color picture after 6 months of specific treatment showing (**a**) an unremarkable posterior pole in the right eye and regression of the subretinal lesion achieving the appearance of atrophy in the (**b**) right and left (**c**) eyes

**Fig. 5:**
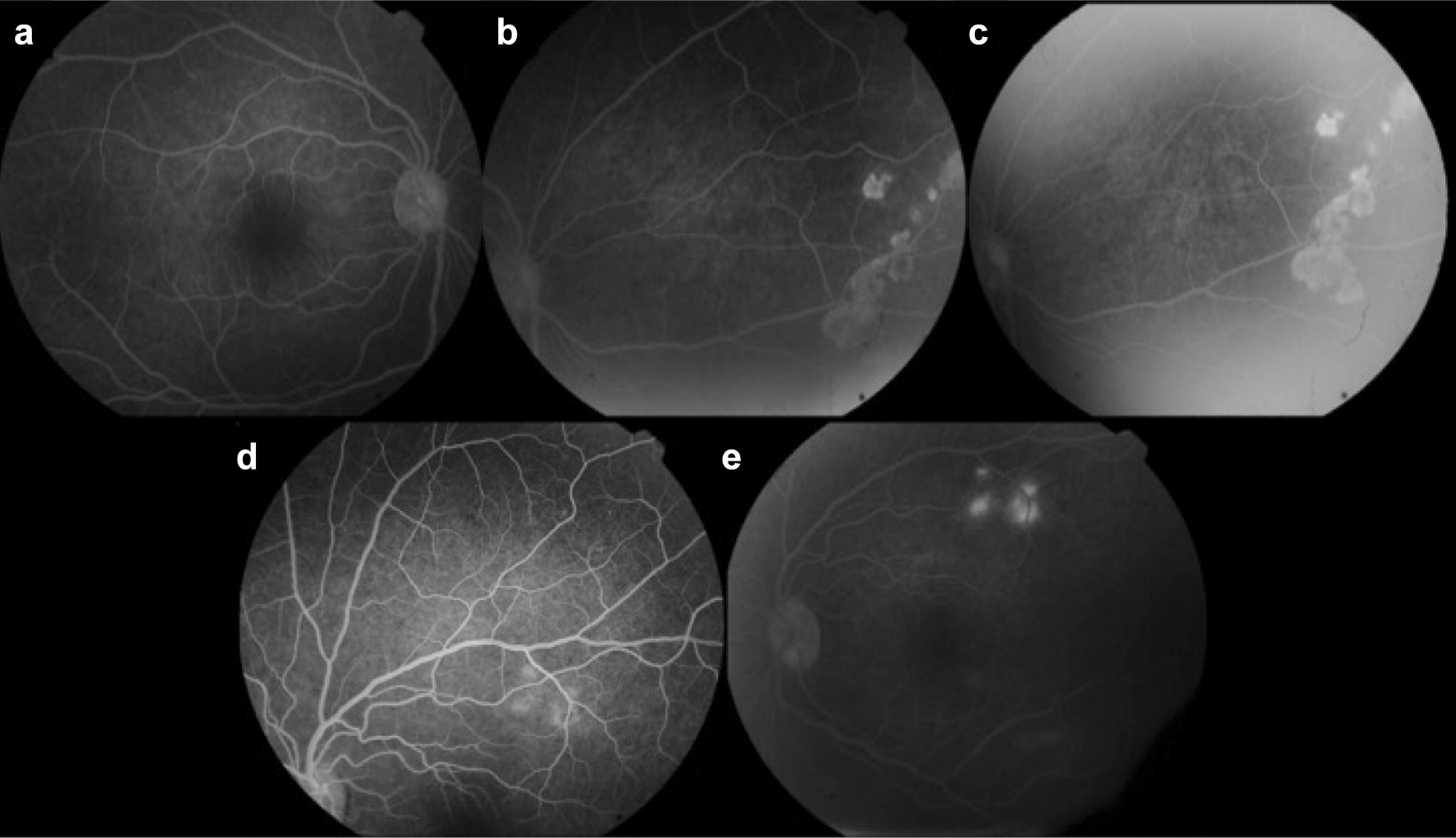
Fluorescein angiography of the right (**a**–**c**) and left eye (**d**, **e**) 6 months after targeted treatment was initiated. The lesions in the right eye showed discrete leakage and areas of window defect and the lesions in the left eye still demonstrated mild leakage

**Fig. 6:**
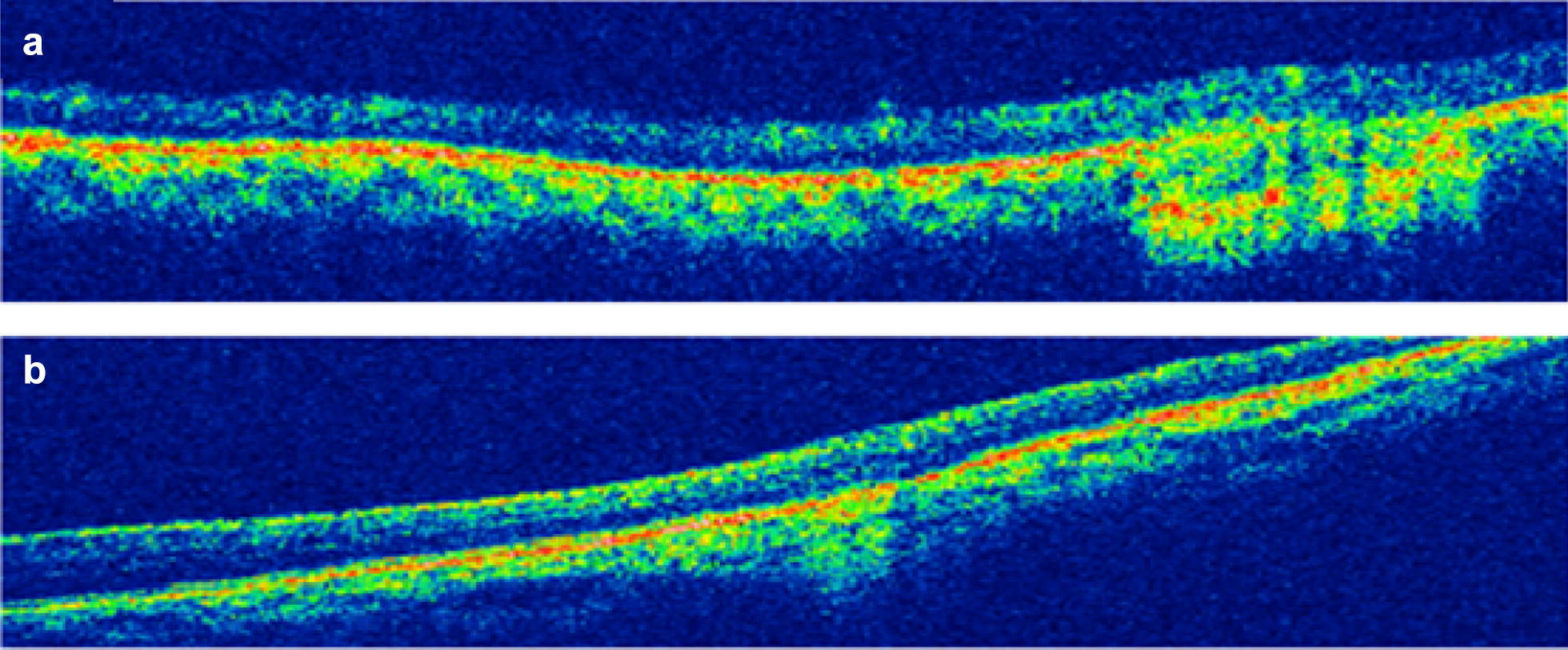
Optical coherence tomography of the lesions of the right (**a**) and left (**b**) eye after 6 months of specific treatment demonstrating the flattening of the subretinal lesions in both eyes with regression of the subretinal fluid and the resulting areas of atrophy

After 6 months of targeted treatment, the fundus exam revealed a significant regression of the lesions (Fig. 4). The FA still showed discrete leakage (Fig. 5) and the OCT demonstrated regression of the choroidal lesion and subretinal fluid, which were replaced by areas of retinal atrophy (Fig. 6).

## Discussion and conclusions

Brazil is an endemic area for tuberculosis [6]. Considering the endemic environment, the chronic immunosuppression, systemic and the retinal findings, treatment for tuberculosis was started, without success. This case reports an immunosuppressed patient by the chronic and irregular use of steroids, developing systemic atypical mycobacteriosis caused by *Mycobacteria Fortuitum* and associated choroidal granuloma.

*Mycobacterium fortuitum* is a ubiquitous organism, often acquired from environmental sources such as water, soil, and dust [7]. It has already been isolated in dairy products, cold-blooded animals, vegetation and human feces [8]. The typical clinical presentation includes cutaneous and soft tissue infections, as happened in our case [5, 7]. It is increasingly recognized as an opportunistic pathogen that causes disseminated infection, especially in patients with impaired immunity or receiving glucocorticoid therapy [7]. A recent review showed that the use of steroids was implicated as a risk for ocular NTM infection in 42.9% of the cases [2]. The risk of NTM increases with oral prednisone doses > 15 mg and immunocompromised patients are more likely to develop intraocular NTM infections [9–11], which are associated with a greater risk of infection of the intraocular tissues, ocular surface, periocular and ocular attachments leading to blindness in the severest cases [2, 5]. In general, the underlying mechanism that increases the risk of NTM disease in immunosuppressed patients is the rupture or depletion of cell-mediated immunity, a critical component of host defense against mycobacteria [5]. There are four groups of NTM and *M. Fotuitum* belongs to group IV, the rapid growers, one of the most commonly affecting the eye. They usually do not respond to conventional anti-tuberculosis treatment and require a prolonged therapy with multiple antibiotics [12]. Sharma et al. reported a subretinal massive lesion caused by infection of three agents: *M. Tuberculosis, M. fortuitum* and *M. Bovis* in an immunosuppressed patient. Their case progressed to phthisis despite treatment [4]. In our case, the focal granulomas were extramacular and fortunately the vision could be preserved with proper treatment.

The occurrence of disseminated infection due to an organism that is not normally virulent requires deep investigation in immunocompromised states [13]. NTM infections are difficult to identify. Despite being fast growing mycobacteria, a high rate of negative cultures commonly cause delays in treatment [1]. Its course is indolent, additionally prolonged with the use of corticosteroids and often refractory to various medical therapies and surgical interventions [2]. In this case, several initial cultures were negative and the patient was treated empirically first with wide spectrum antibiotics, then for presumptive systemic tuberculosis. *M. fortuitum* was isolated only after 45 days of investigation.

Gold standard therapy for *M. fortuitum* has not been established by clinical trials [3, 14]. The pathogen is usually non responsive to conventional anti tuberculous treatment however it is susceptible to several oral antibiotics, such as the newer macrolides, quinolones, and sulfonamides [15]. A combination of antibiotics based on culture sensitivities is recommended in order to decrease the likelihood of resistance developing [2]. A typical choroidal granuloma caused by *M. fortuitum* is a rare presentation of the infection and our report showed a good outcome with proper treatment.

## Data Availability

All data generated or analysed during this study are included in this published article.

## Abbreviations

NTM: nontuberculous mycobacteria
FA: fluorescein angiography
OCT: optical coherence tomography
RPE: retinal pigment epithelium
